# Cadmium-Related Mortality and Long-Term Secular Trends in the Cadmium Body Burden of an Environmentally Exposed Population

**DOI:** 10.1289/ehp.11667

**Published:** 2008-07-24

**Authors:** Tim S. Nawrot, Etienne Van Hecke, Lutgarde Thijs, Tom Richart, Tatiana Kuznetsova, Yu Jin, Jaco Vangronsveld, Harry A. Roels, Jan A. Staessen

**Affiliations:** 1 Studies Coordinating Centre, Division of Hypertension and Cardiovascular Rehabilitation, Department of Cardiovascular Diseases; 2 School of Public Health, Department of Occupational and Environmental Medicine, University of Leuven, Leuven, Belgium; 3 Department of Environmental Biology, University of Hasselt, Diepenbeek, Belgium; 4 Social and Geography Section, Department of Earth and Environment Sciences, University of Leuven, Leuven, Belgium; 5 Department of Epidemiology, Maastricht University, Maastricht, the Netherlands; 6 Industrial Toxicology and Occupational Medicine Unit, Université catholique de Louvain, Brussels, Belgium

**Keywords:** cadmium, environmental exposure, mortality

## Abstract

**Background:**

Few population studies have reported on the long-term changes in the internal cadmium dose and simultaneously occurring mortality.

**Objective:**

We monitored blood cadmium (BCd), 24-hr urinary cadmium (UCd), and mortality in an environmentally exposed population.

**Methods:**

Starting from 1985, we followed BCd (until 2003), UCd (until 1996), and mortality (until 2007) among 476 and 480 subjects, randomly recruited from low- exposure areas (LEA) and high-exposure areas (HEA). The last cadmium-producing plant in the HEA closed in 2002.

**Results:**

From 1985–1989 to 1991–1996, BCd decreased by 40.3% and 18.9% in the LEA and HEA, respectively (*p* < 0.0001 for between-area difference). From 1991–1996 until 2001–2003, BCd remained unchanged in the HEA (+ 1.8%) and increased by 19.7% in the LEA (*p* < 0.0001). Over the entire follow-up period, the annual decrease in BCd averaged 2.7% in the LEA (*n* = 258) and 1.8% in the HEA (*n* = 203). From 1985–1989 to 1991–1996, UCd fell by 12.9% in the LEA and by 16.6% in the HEA (*p* = 0.22), with mean annual decreases of 2.7% (*n* = 366) and 3.4% (*n* = 364). Over 20.3 years (median), 206 deaths (21.5%) occurred. At baseline, BCd (14.6 vs. 10.2 nmol/L) and UCd (14.1 vs. 8.6 nmol/24-hr) were higher in deaths than in survivors. The risks (*p* ≤ 0.04) associated with a doubling of baseline UCd were 20% and 44% for total and noncardiovascular mortality, and 25% and 33% for a doubling of BCd.

**Conclusions:**

Even if zinc–cadmium smelters close, historical environmental contamination remains a persistent source of exposure. Environmental exposure to cadmium increases total and noncardiovascular mortality in a continuous fashion without threshold.

Cadmium is a metal with high toxicity, has an estimated elimination half-life of 10–30 years, and accumulates in the human body, particularly in the liver and the kidney (Järup et al. 1983; Nordberg et al. 2007). Urinary excretion of cadmium over 24 hr (UCd) is a biomarker of lifetime exposure, whereas the blood cadmium concentration (BCd) reflects recent exposure over months (Nordberg et al. 2007). Exposure to cadmium occurs through intake of contaminated food or water or by inhalation of tobacco smoke or polluted air (Hogervorst et al. 2007; Nordberg et al. 2007). Environmental exposure to cadmium in northeastern Belgium, in the neighborhood of zinc–cadmium smelters, has been associated with a nearly 30% increased urinary cadmium excretion (Sartor et al. 1992b), renal dysfunction (Buchet et al. 1990; Staessen et al. 1994), increased calciuria (Staessen et al. 1991b), osteoporosis (Staessen et al. 1999), a 35% population-attributable risk of fractures (Staessen et al. 1999), and a 67% population-attributable risk of lung cancer (Nawrot et al. 2006).

Studies of Japanese populations living in areas heavily polluted by cadmium have shown that the cadmium-induced renal tubular injury (Arisawa et al. 2001, 2007a, 2007b; Nakagawa et al. 2006; Nishijo et al. 2004, 2006; Uetani et al. 2006), even in the presence of moderate elevations of the urinary β_2_-microglobulin excretion (300–1,000 μg/g creatinine), adversely affected life prognosis. To our knowledge, no cohort studies in a general population have reported on long-term changes in the body burden of cadmium and the simultaneous incidence of mortality. In our environmentally exposed cohort, living in Belgium, we monitored BCd from 1985 until 2003, urinary cadmium from 1985 until 1996, and mortality from 1985 to 2007. During this period, cadmium emissions ceased, but the soil remained contaminated with cadmium. Our primary objective was to assess the association between mortality and the internal dose of cadmium. We also evaluated how attrition by cadmium-related mortality (Arisawa et al. 2007b) might affect recent estimates of the internal cadmium dose.

## Materials and Methods

### Study population

**Figure 1:**
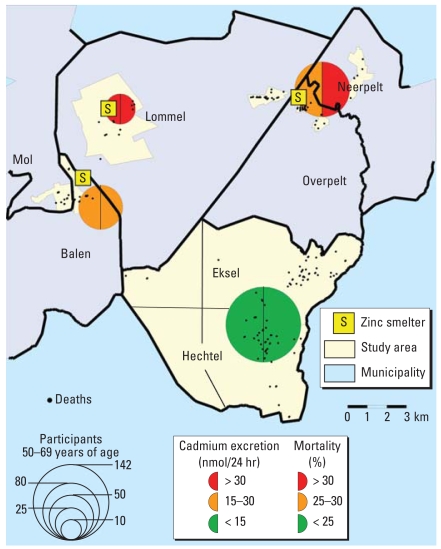
Geographic representation of UCd at baseline (1985–1989) and spatial analysis of mortality up to 30 September 2007, in participants 50–69 years of age at enrollment. Dots represent the homes of the deceased participants. The diameters in the lower left diagram represent the number of participants per area.

The Flemish participants enrolled in the Cadmium in Belgium study (CadmiBel) were recruited from September 1985 through December 1989 from northeastern Belgium (Noorderkempen). This region has an area contaminated with cadmium and a reference area with lower exposure to cadmium (Figure 1). We selected 10 districts where we expected the mean concentration of cadmium in the soil to be more than 3 mg/kg [high-exposure area (HEA)] as opposed to < 1 mg/kg [low-exposure area (LEA)] on the basis of a preliminary screen done in 1983–1984 by the Research Institute for Ecology and Forestry, Genk, Belgium. The HEA of 300 km^2^ has an estimated population of 9,840, borders on three zinc–cadmium smelters, and consists of six districts of the municipalities Mol, Balen, Lommel, Overpelt, and Neerpelt (Figure 1). This area remains polluted by cadmium, despite the dismantlement of the smelter in Lommel in 1974, the transition from pyrolytic to electrolytic zinc refining in Overpelt in the 1970s, and a complete cessation of the cadmium production in Overpelt in 1992 and in Balen in 2002. The reference area has 9,390 inhabitants, is located > 10 km southeast of the smelters, and includes four districts of the villages Hechtel and Eksel (Figure 1).

**Figure 2:**
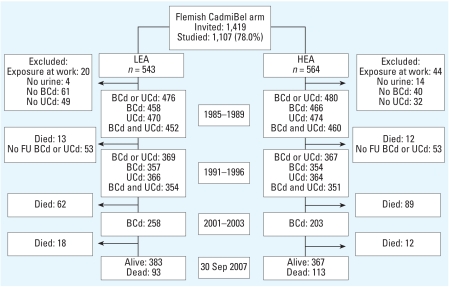
Flow chart of the Flemish cohort from 1985–1989 until 2007. FU, follow-up.

In every district, we identified a random population sample stratified by sex and age (20–39 years vs. 40–59 years vs. ≥ 60 years), with the aim of recruiting equal numbers in each stratum. The municipalities gave listings of all inhabitants sorted by address. Households, defined as those who lived at the same address, were the sampling unit. We numbered households consecutively and generated a random number list by use of a SAS random function (version 9.1.3; SAS Institute Inc., Cary, NC, USA). Households with a number matching the list were invited. Household members > 20 years of age were eligible but were not included if the quota of an age–sex stratum had been met. Of 1,419 invited subjects, 1,107 participated (78.0%) (Figure 2). We complied with all applicable requirements of U.S. and international regulations, particularly the Helsinki Declaration, for investigation of human subjects. The Ethics Review Board of the Medical Faculty of the University of Leuven approved the study. Participants gave informed consent at recruitment and renewed consent at follow-up.

At baseline (1985–1989), every household was repeatedly visited by the same study nurse, who gave participants a self-administered questionnaire and a container for urine sampling. If needed, nurses assisted participants in completion of the questionnaire. They instructed participants on how to obtain 24-hr urine samples without external contamination. The questionnaire inquired about lifestyle, past and current residence, possible exposure to cadmium at work, smoking habits, and previous medical history. For the present analysis, we classified subjects who smoked at enrollment as smokers. One week after the first home visit, nurses revisited the homes to collect the questionnaire and the 24-hr urine samples and to obtain a sample of venous blood. The nurses measured anthropometric characteristics and obtained from each subject five consecutive blood pressure readings. They used the same procedures at follow-up. For participants reporting possible exposure to cadmium at work, the occupational-health physician of the company that owned the plants gave details on their employment history and their role in the production process. In keeping with previous publications (Nawrot et al. 2006), we excluded from our main analyses (Figure 2) 42 smelter workers with documented exposure at work (3 in LEA vs. 39 in HEA) and 22 coal miners with documented pneumoconiosis (17 vs. 5).

The principal investigator (J.A.S.) coordinated the administration of questionnaires and wrote the manuals of operation, code books, and SAS programs for compilation of the coded data sheets. He developed the programs to convert questionnaire replies and codes into analyzable variables. Questionnaires were coded by trained nurses. Technicians entered the data into a SAS database. For quality assurance, we randomly selected 10% of questionnaires coded by a nurse and had them recoded by another nurse. We input all data twice into the database by different technicians. We compared duplicate data sets with the PROC COMPARE application in the SAS software to trace input errors. Data coders and SAS programs checked internal consistency of questionnaire replies. We coded socioeconomic status (SES) according to the methods of the U.K. Office of Population Censuses and Surveys (1980) and condensed it into a scale with scores ranging from 1 to 3 (Staessen et al. 1994, 1999).

### Measurement of cadmium

Participants collected urine samples obtained over 24 hr in a wide-neck polyethylene container. We measured BCd and UCd with an electrothermal atomic absorption spectrometer fitted with a stabilized-temperature-platform furnace and Zeeman background correction. In an external quality-control program completed by Trace Element Control Scheme (organized by Robens Institute, University of Surrey, Guildford, UK), the accuracy of the cadmium measurements did not differ significantly over time (Claeys et al. 1992; Staessen et al. 2000).

### Biochemical measurements

At baseline (1985–1989), using methods described elsewhere (Lauwerys et al. 1990), we measured the serum concentration of creatinine (SCrt) as index of glomerular renal function, the serum levels of high-density lipoprotein (HDL) and total cholesterol, the activity in serum of γ-glutamyltransferase as index of alcohol intake, and the urinary excretion of creatinine and retinol-binding protein (RBP) as indicators of the completeness of the urine collection and renal tubular function, respectively. For statistical analysis, we set the urinary measurements to missing if, according to previously published criteria (Staessen et al. 1983), the 24-hr urine sample was incomplete or overcollected.

### Assessment of mortality

Via the National Population Registry (Rijksregister) in Brussels, Belgium, we ascertained the vital status of all participants until 30 September 2007. We obtained the *International Classification of Diseases, 9th Revision* (2007) codes for the immediate and underlying causes of death from the Flemish Registry of Death Certificates (Brussels, Belgium). We checked diseases reported on death certificates systematically against records held by general practitioners, hospitals, or both.

### Spatial analyses

We located participants’ houses and zinc–cadmium smelters by use of the global positioning system GPS Pathfinder Pro XL (Trimble Navigation Europe, Hook, Hampshire, UK). We converted degrees longitude and latitude (projection of the earth’s curved surface onto a flat map by use of ellipsoid WGS84) into kilometers by use of the Lambert projection system of Belgian maps. We used SAS/GRAPH mapping software and the database of TeleAtlas (Gent, Belgium). We calculated spatial summary statistics for small geographic sectors (Figure 1), consisting of one or two statistical units as defined by the Belgium National Institute of Statistics.

### Statistical methods

For database management and statistical analyses, we used SAS software, version 9.1.3 (SAS Institute Inc.). We log-transformed non-normally distributed data and report these results as geometric mean and interquartile range (IQR). We compared means using the large-sample *Z*-test, medians using Mann–Whitney’s test, and proportions using Fisher’s exact test. We investigated associations between variables by use of simple and multiple linear regression. We applied Cox regression to model the relation between failure time (occurrence of fatal events) and BCd and UCd, while adjusting for significant covariables (sex, age, body mass index) and lifestyle (smoking status, γ-glutamyltransferase activity in serum as index of alcohol intake) and SES. We checked the proportional hazards assumption by the Kolmogorov-type supremum test, as implemented in the PROC PHREG procedure of the SAS package, and by testing the interaction terms between follow-up duration and the internal dose of cadmium. In stepwise regression, we set *p*-values for variables to enter and stay in the models at 0.15. Where appropriate, we adjusted our analyses for extra variables, such as sex, body mass index, systolic blood pressure, and the ratio of HDL to total cholesterol in serum. All *p*-values were for two-sided tests.

## Results

### Characteristics of subjects

At baseline (1985–1989), 476 residents of the LEA and 480 subjects of the HEA had a measurement of their BCd or UCd; 452 and 460 participants, respectively, had both measurements (Figure 2). At baseline (1985–1989) and at the first follow-up examination (1991–1996), the characteristics of the residents of the districts near the smelters did not differ from those living in the LEA except for BCd, UCd, and distance to the nearest smelter (Table 1). The 24-hr urinary excretion of RBP at baseline was significantly lower in the LEA compared with that in the HEA (Table 1). At baseline, in the LEA, the number of participants with low, intermediate, or high SES was 355 (74.5%), 115 (24.2%), and 6 (1.3%), respectively, whereas in the HEA, these numbers were 410 (85.4%), 69 (14.4%), and 1 (0.2%). The *p*-value for the between-area difference in the distribution of SES was < 0.0001. In 2001–2003 (Table 1), the BCd still tended to be lower in the LEA than in the HEA (7.4 vs. 8.2 nmol/L; *p* = 0.059).

Of 476 residents of the LEA and 480 subjects of the HEA who had a measurement of their BCd or UCd at baseline (1985–1989), in each area, 53 did not undergo a follow-up measurement of their internal dose of cadmium. Compared with participants who had at least one such measurement during follow-up, they had largely similar characteristics (Table 2).

### Cohort analyses of the internal dose of cadmium

**Figure 3:**
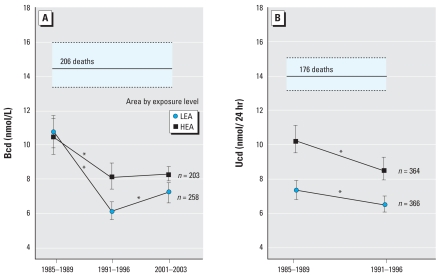
BCd (*A*) and UCd (*B*) in the cohort analysis. The horizontal bar (deaths) indicates the geometric mean (solid line) with 95% CI (dashed lines) of the internal dose in subjects who died before 30 September 2007 (significance of the difference with survivors, *p* < 0.0001). *Significant difference between consecutive measurements.

The cohort with BCd available at three time points included 258 residents of the LEA and 203 of the HEA (Figure 2). Median follow-up from the first to the third measurement of BCd amounted to 13.2 years (IQR, 11.5–14.7; 5th–95th percentile interval, 8.2–16.4 years) and 15.9 years (IQR, 9.8–16.8; 5th–95th percentile interval, 9.2–17.8 years), respectively. Figure 3A illustrates the geometric mean levels of BCd over time by study area. On an individual basis, from 1985–1989 to 1991–1996, BCd decreased by 40.3% [95% confidence interval (CI), 36.3 to 44.9%; *p* < 0.0001] in the LEA and by 18.9% (95% CI, 14.7 to 22.9%; *p* < 0.0001) in the HEA (*p* < 0.0001 for between-area difference). From 1991–1996 until 2001–2003, BCd did not change in the HEA (+1.8%; 95% CI, –4.2 to 8.0%; *p* = 0.96) and increased by 19.7% (95% CI, 13.2 to 26.6%; *p* < 0.0001) in the reference area (*p* < 0.0001 for between-area difference). Over the whole follow-up period, the annual decrease in the BCd averaged 2.7% (95% CI, 2.3 to 3.4%) in the reference area and 1.8% (95% CI, 1.4 to 2.3%) in the contaminated area.

The cohort with measurements of UCd at baseline (1985–1989) and follow-up (1991–1996) consisted of 366 residents of the reference area and 364 of the area close to the smelters. Median follow-up from the first to the second measurement of UCd amounted to 4.96 years (IQR, 4.63–6.00; 5th–95th percentile interval, 4.54–7.09 years) in the LEA and to 5.24 years (IQR, 5.08–5.33; 5th–95th percentile interval, 4.60–5.50 years) in the HEA. Figure 3B gives the geometric mean levels of the UCd over time by study area. On an individual basis, the UCd fell from 1985–1989 to 1991–1996 by 12.9% (95% CI, 9.0 to 16.8%; *p* < 0.0001) in the LEA and by 16.6% (95% CI, 12.3 to 20.7%; *p* < 0.0001) in the HEA (*p* = 0.22 for between-area difference). The annual decrease in the UCd averaged 2.7% (95% CI, 1.8 to 3.6%) in the reference area and 3.4% (95% CI, 2.5 to 4.3%) in the contaminated area.

### Determinants of the internal dose at follow-up

Table 3 gives the determinants of the BCd in 1991–1996 and in 2001–2003. The internal dose of cadmium was not significantly related to sex or body mass index. BCd at follow-up increased with higher baseline values (1985–1989), age, smoking, and shorter distance to the nearest smelter. BCd at follow-up was also inversely and independently correlated with the serum ferritin concentration at baseline. In quantitative terms, BCd at follow-up rose by approximately 52% for a doubling of the cadmium level at baseline (1985–1989), by approximately 12% for each 10-year increase in age at enrollment, and by approximately 22% in smokers, but decreased by approximately 16% for a doubling in the distance to the nearest smelter, and by approximately 8% for a 2-fold increase in the serum ferritin concentration. The determinants of UCd at follow-up (1991–1996) were the same as for BCd. Quantitatively, UCd at follow-up rose by approximately 58% for a doubling of the cadmium excretion at baseline (1985–1989), by approximately 7% for each 10-year increase in age, and by approximately 18% in smokers, but it decreased by approximately 2% for a doubling in the distance to the nearest smelter and by approximately 3% for a 2-fold increase in the baseline serum ferritin concentration.

In analyses limited to premenopausal women, similarly adjusted as in Table 3 (excluding the sex term), the partial regression coefficients for serum ferritin were −0.114 ± 0.047 log pmol/L (*p* = 0.015) and −0.159 ± 0.042 log pmol/L (*p* < 0.001) for BCd in 1991–1996 and 2001–2003, respectively, and −0.051 ± 0.038 log pmol/L (*p* = 0.18) for UCd in 1991–1996.

### Analysis of mortality

As shown by exposure area in Table 2, subjects who died, compared with survivors, had higher baseline values of age (66.6 vs. 41.8 years; *p* = 0.0001), systolic blood pressure (141.5 vs. 124.9 mmHg; *p* < 0.0001), diastolic blood pressure (78.2 vs. 74.7 mmHg; *p* = 0.0001), and serum total cholesterol (6.44 vs. 5.78 mmol/L; *p* < 0.0001) and lower serum HDL cholesterol (1.17 vs. 1.29 mmol/L; *p* < 0.0001). Subjects who died had a higher BCd (*n* = 195; 14.6 vs. 10.2 nmol/L; *p* < 0.0001 ) and a higher UCd (*n* = 201; 14.1 vs. 8.6 nmol/24-hr; *p* < 0.0001) than those who survived until 30 September 2007. This was also the case (Figure 1) when the analysis was limited to subjects 50–69 years of age at baseline (BCd: *n* = 75; 14.2 vs. 11.9 nmol/L; *p* = 0.037; UCd: *n* = 81; 16.8 vs. 13.3 nmol/24-hr; *p* = 0.005).

In all subjects with an assessment of the internal dose of cadmium at baseline, median follow-up of vital status amounted to 20.3 years (IQR, 18.8–20.8; 5th–95th percentile interval, 4.9–21.9 years). Over this period, 206 deaths occurred (Table 4). The cause of death was cardiovascular in 88, noncardiovascular in 96, suicidal or accidental in 5 subjects, and unknown in 17 participants. Noncardio-vascular mortality included 54 cancers (17 lung cancers). With adjustments applied for sex, age, body mass index, smoking, γ-glutamyl-transferase as index of alcohol intake, and SES, the risk of all-cause and noncardiovascular mortality and the risk of death from all cancers and lung cancer increased with higher UCd. The risk increments associated with a doubling of UCd amounted to 20% and 44% for total and noncardiovascular mortality, and to 43% and 62% for total and lung cancer mortality (Table 4). With similar adjustments applied, the risk increments associated with a doubling of the BCd were 25% and 33% for total and noncardiovascular mortality (Table 4). The interaction terms between sex and the internal dose of cadmium did not reach statistical significance (*p* ≥ 0.27).

**Figure 4:**
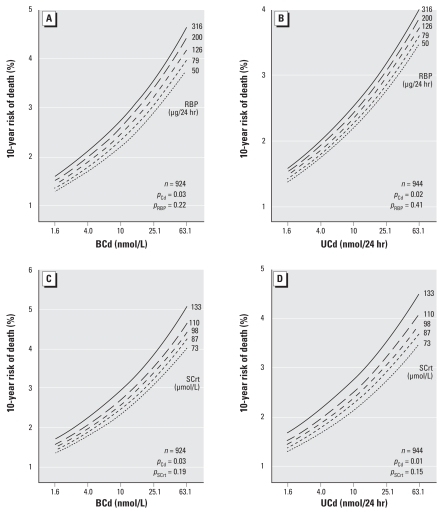
Ten-year risk of death in relation to the BCd (*A* and *C*) and UCd (*B* and *D*) at baseline with standardization to the distribution (mean or ratio) of sex, age, body mass index, smoking, γ-glutamyltransferase as index of alcohol intake, and SES: risk function for the 5th, 25th, 50th, 75th, and 95th percentiles of the 24-hr urinary excretion of RBP (*A* and *B*) and the SCrt (*C* and *D*). *p*-Values are for the independent effects of cadmium (*p*_Cd_), urinary RBP (*p*_RBP_), and serum creatinine (*p*_SCrt_). The range of the internal cadmium dose plotted along the horizontal axes corresponds with the 1st to 99th percentile of BCd and UCd.

Figure 4 illustrates the 10-year risk of death in relation to the BCd and UCd at baseline with standardization to the distribution (mean or ratio) of sex, age, body mass index, smoking, γ-glutamyltransferase, and SES. We plotted the risk functions for the 5th, 25th, 50th, 75th, and 95th percentiles of the 24-hr urinary excretion of RBP and the SCrt, as indicators of tubular and glomerular renal function, respectively. These analyses show continuous and significantly positive associations between all-cause mortality and the internal cadmium dose (*p* ≤ 0.03), whereas in the presence of cadmium the associations with the indexes of renal function were not significant (*p* ≥ 0.15).

Sensitivity analyses of mortality in relation to UCd at baseline produced results that were not materially different from those in Table 4. After additional adjustment for systolic blood pressure and the ratio of HDL to total cholesterol, the hazard ratios were 1.20 (95% CI, 1.03–1.39; *p* = 0.018) for total mortality; 1.06 (95% CI, 0.84–1.33; *p* = 0.65) for cardiovascular mortality; 1.41 (95% CI, 1.14–1.73; *p* = 0.002) for noncardiovascular mortality; 1.45 (95% CI, 1.17–1.79; *p* = 0.0007) for all cancers; and 1.60 (95% CI, 0.99–2.56; *p* = 0.051) for lung cancer. After exclusion of 5 violent deaths and 17 deaths of unknown cause, the adjusted hazard ratio for all-cause mortality in relation to UCd was 1.19 (95% CI, 1.01–1.37; *p* = 0.035). Analyses in which we additionally included 42 smelter workers were confirmatory (Table 5).

Figure 1 illustrates the spatial association between all-cause mortality and the baseline UCd in subjects who were 50–69 years of age at enrollment (1985–1989). Mortality clustered around the industrial settlements in Lommel and Overpelt, and it was associated with higher cadmium body burden. For contrasting subjects with a UCd < 15 nmol and > 30 nmol (Figure 1), with adjustments applied for sex, age, and smoking, the attributable (etiologic) fraction and the population-attributable fraction of all-cause mortality were 48.5% and 9.4%, respectively.

## Discussion

The key finding of our study was that in exclusively environmentally exposed subjects, the internal dose of cadmium predicted total and noncardiovascular mortality. As reported previously (Nawrot et al. 2006), UCd also predicted lung cancer. These findings were consistent in analyses also including 42 smelter workers. Total mortality includes cardiovascular mortality. Subclinical cardiovascular disease can aggravate the course of noncardiovascular diseases. This provided the rationale for running Cox models additionally adjusted for cardiovascular risk factors, such as systolic blood pressure and the ratio of HDL to total cholesterol in serum. These more fully adjusted models produced confirmatory results. To focus on premature mortality, in our spatial analyses we considered total mortality in relation to UCd in subjects 50–69 years of age at enrollment. Mortality clustered around the industrial settlements in Lommel and Overpelt, and it was associated with higher cadmium body burden.

Our present findings are in line with previous studies in Japanese populations, which showed association between mortality and environmental exposure to cadmium (Arisawa et al. 2001, 2007a, 2007b; Nakagawa et al. 2006; Nishijo et al. 2004, 2006; Uetani et al. 2006). However, there are also important differences between our present results and the observations in Japanese. First, the median UCd level in Japanese was 7.0 μg/g creatinine (Arisawa et al. 2007b), which probably explains the increased mortality from nephritis and nephrosis (Nakagawa et al. 2006; Nishijo et al. 2006). By comparison, in our study, the median UCd at baseline (1985–1989) was 0.74 μg/g and 1.03 μg/g creatinine, in the LEA and HEA districts, respectively. In Swedish postmenopausal women, the urinary concentration was 0.67 μg/g creatinine (Åkesson et al. 2006). Second, in the Japanese studies (Arisawa et al. 2001, 2007a, 2007b; Nakagawa et al. 2006; Nishijo et al. 2004, 2006; Uetani et al. 2006), exposure occurred mainly via consumption of contaminated rice and rice derivatives. In our cohort, a 2-fold increase in the metal loading rate in house dust was associated with increases (*p* < 0.001) in BCd (+2.3%) and UCd (+3.0%), independent of the intake of locally grown vegetables (Hogervorst et al. 2007). These findings highlight that in our cohort, contaminated house dust was a persistent source of exposure. Moreover, the emissions from the zinc–cadmium smelters were approximately 1,000 times higher in the period 1950–1980 than in the 1990s (Staessen et al. 1995). Inhalation of contaminated particulate matter, with the lungs being both the route of entrance and the target organ, explains why in contrast to the Japanese studies (Arisawa et al. 2007a), we found a positive and independent association between the risk of lung cancer and lifetime exposure, as reflected by UCd. Third, in the Japanese studies, the mortality associated with exposure was mainly cardiovascular with greater risks of heart failure and cerebral infarction (Nishijo et al. 2006). In our cohort, the association of cardiovascular mortality with the internal cadmium dose was nonsignificant. A 2-fold increase in UCd was even associated with a 30–40% decrease in cerebrovascular mortality. Blood pressure is the most consistent and powerful predictor of stroke (Zhang et al. 2006). We previously demonstrated an inverse and independent association between blood pressure and the internal cadmium dose in men (Staessen et al. 1991a). Recently, we also noticed that a higher cadmium body burden was associated with lower pulse pressure throughout the arterial system, lower aortic pulse wave velocity, and higher femoral distensibility (Schutte et al. 2008). Finally, Japanese researchers reported that cadmium exposure aggravated mortality more in women than men (Nishijo et al. 2004; Uetani et al. 2006), whereas in our Cox models the interaction terms between sex and the internal cadmium dose did not reach significance for any mortality end point.

Most Japanese studies used as biomarkers of exposure either microproteinuria (Arisawa et al. 2007b; Nishijo et al. 2004) or other indexes of tubular renal dysfunction (Arisawa et al. 2007a, 2007b; Nishijo et al. 2004, 2006), which were analyzed as categorical variables. Fewer Japanese studies presented the urinary cadmium-to-creatinine ratio in categorical analyses as biomarker of the internal cadmium dose (Nakagawa et al. 2006). We analyzed BCd and UCd as continuous variables. In Cox models including the internal cadmium dose as well as the 24-hr urinary excretion of RBP or the SCrt as indexes of tubular and glomerular renal function, respectively, only cadmium was a significant predictor of mortality. These findings suggest that the increased mortality was directly related to the toxic effects of cadmium, rather than being mediated by renal dysfunction, as suggested by the Japanese studies (Arisawa et al. 2007a, 2007b; Nishijo et al. 2004, 2006).

From 1985–1989 until 1991–1996, the BCd decreased on average by approximately 40% and 20% in the LEA and HEA, respectively. Over the same interval, UCd decreased by approximately 15%. Only 106 participants (~ 11.0%) had no follow-up measurement of BCd or UCd. Nonparticipants had similar characteristics as those with follow-up measurements in 1991–1996 and/or 2001–2003. Three mechanisms explain the observed decline in the internal cadmium dose. First, changes in the industrial activity certainly contributed. In the study region, zinc smelters had emitted cadmium into the atmosphere since 1888 (Staessen et al. 1994, 1995). In the 1970s, the zinc ovens were replaced by electrolytic refining, such that the annual airborne cadmium emissions dropped from 125,000 kg in 1950 to 130 kg in 1989 (Staessen et al. 1995). In 1992, the primary zinc smelter in Overpelt ceased activity (Buchet et al. 1996). In Lommel and Overpelt, the daily cadmium dustfall fell from 12 mg/m^2^ in 1985 to < 0.01 mg/m^2^ in 1994 (Staessen et al. 1995). Second, the inhabitants of the contaminated area were informed, for the first time in a systematic way around 1995, how to reduce their environmental exposure to cadmium by using tap water instead of well water for drinking and cooking, by liming the soil of their kitchen gardens, and by not eating locally grown leafy vegetables (Staessen et al. 1995). Third, at baseline, deceased participants had substantially higher BCd and UCd than those who survived. As first suggested by Japanese studies (Arisawa et al. 2007b), these findings underscore that attrition of the original cohort by cadmium-related mortality contributed to the apparent decrease in the internal cadmium dose in our cohort.

From 1991–1996 until 2001–2003, the BCd did not show any further decrease in the contaminated area. Cadmium emitted in the past continues to contaminate the soil not only in the HEA, but also in the nearby reference area. Polluted house dust is a persistent source of exposure to cadmium (Hogervorst et al. 2007; Kimbrough et al. 1994; Paustenbach et al. 1997). In general, the concentrations of contaminants are greater in house dust compared with exterior soil. For instance, in a review of 15 studies, the median of the concentration ratios of lead in house dust versus external soil was 2.3 (Paustenbach et al. 1997). Fine dust particles are mobile, adhere easily to the skin (Kissel et al. 1996), and enhance the bioavailability of the contaminants that they carry (Kissel et al. 1996; Paustenbach et al. 1997). Preventive measures that diminish exposure to house dust, such as hand washing and dust control indoors, are now being recommended in the polluted area, but not in the LEA (Hogervorst et al. 2007). Residents of the reference area perceive the probability of exposure to cadmium as low and are therefore less likely to implement preventive measures. Interestingly, residents of the LEA had higher UCd than did inhabitants of another cadmium-polluted area of Belgium, the city of Liège (Sartor et al. 1992a). A combination of these factors might explain the approximately 20% increase in the BCd in the LEA from 1991–1996 until 2001–2003.

In humans, after cessation of long-term high exposure, the decrease in BCd displays a slow component with a half-life of 7–16 years and a fast component with a half-life of 3–4 months (Nordberg et al. 2007). The biologic half-life of cadmium in the kidney is in the order of 20 years (Nordberg et al. 2007). As expected, the baseline levels of the biomarkersof cadmium exposure were the main determinant of their value at follow-up. Throughout follow-up, cadmium levels remained higher in smokers and older subjects than in nonsmok-ers and younger people. They decreased with greater distance to the nearest smelter and with higher body iron stores. Iron deficiency stimulates the gastrointestinal absorption of cadmium (Flanagan et al. 1978). Our findings are in line with a study of nonsmoking Swedish farmers (Olsson et al. 2002). Because of iron deficiency, women had a 40% higher BCd and a 60% higher urinary cadmium-to-creatinine ratio than men. In our present study, sensitivity analyses confined to pre-menopausal women showed associations similar as in the whole study population between the internal cadmium dose at follow-up and the serum ferritin concentration at baseline.

The present study must be interpreted within the context of its potential limitations and strengths. First, there was substantial overlap in the distributions of BCd and UCd between the residents of the LEA and HEA. This explains why the separation between the two study areas in terms of mortality rates appears smaller than expected. Epidemiologic studies, using aggregate data, are prone to ecologic biases (Järup and Best 2003), while attempting to deduce individual-level effects from group-level data. Relating biomarkers of effect and exposure at the individual level is key to detect true associations (Hill 1965; Järup and Best 2003). The relations we observed here between mortality and internal cadmium dose, based on individual data, satisfy Hill’s criteria for causality (Hill 1965; Nawrot et al. 2007). Second, we did not measure UCd in 2001–2003, because our environmental research was not funded. In an independent survey commissioned by the Flemish government in 2007 (Wildemeersch 2008), the UCd standardized to mean age (49.6 year) and the proportion of women (51%) and smokers (18%) in the sample (participation rate, 48%) averaged 0.45 μg/g and 0.47 μg/g creatinine in the LEA and HEA, respectively. The corresponding values for the BCd were 4.45 nmol/L and 4.80 nmol/L. For comparison, our results for BCd in 2001–2003, standardized as in the Flemish survey (Wildemeersch 2008), were 6.2 and 6.9 nmol/L in the LEA and HEA, respectively. These findings suggest a steady decrease in the internal cadmium dose, albeit at a much slower pace than from 1985–1989 until 1991–1996.

Our present findings might be relevant for other contaminated areas. For instance, in the United States, ecologic studies demonstrated cadmium pollution not only close to industrial settlements (Gale et al. 2004) or mines (Peplow and Edmonds 2004), but also in agricultural (Schmitt et al. 2006) and coastal (Karouna-Renier et al. 2007) areas. Japanese women remain currently more exposed to cadmium than other rice-dependent populations in Asia (Watanabe et al. 2004). Regulators have to realize that because of its health effects (Buchet et al. 1990; Nawrot et al. 2006; Staessen et al. 1991b, 1994b, 1999) and its very long biologic half-life (Nordberg et al. 2007), environmental exposure to cadmium due to human activities is unacceptable.

In conclusion, environmental exposure to cadmium increases the risk of death. The hazard function is continuous without a threshold of the internal dose below which the risk would disappear. Even if zinc–cadmium smelters cease activity, historical environmental contamination remains a persistent source of exposure. Attrition of cohorts by cadmium-related mortality contributes to the apparent improvement in the measured internal cadmium dose.

## Figures and Tables

**Table 1:** Characteristics of subjects at three consecutive examinations.

	1985–1989		1991–1996		2001–2003	
Characteristic	LEA	HEA	LEA	HEA	LEA	HEA
No.	476	480	369	367	258	203
Women [no. (%)]	260 (54.6)	265 (55.1)	201 (54.5)	206 (56.1)	142 (55.3)	111 (54.7)
Age [years (mean ± SD)]	46. 9 ± 15.4	47.3 ± 15.5	50.0 ± 14.1	49.6 ± 14.5	57.1 ± 13.5	55.2 ± 13.2
Body mass index [kg/m^2^ (mean ± SD)]	25.7 ± 4.2	26.1 ± 4.6	26.0 ± 4.3	26.5 ± 5.1	26.6 ± 4.2	27.1 ± 5.1
Systolic pressure [mmHg (mean ± SD)]	129 ± 18	128 ± 16	126 ± 18	129 ± 19	128 ± 18	127 ± 16
Diastolic pressure [mmHg (mean ± SD)]	75 ± 9	76 ± 9	78 ± 10	79 ± 11	77 ± 10	77 ± 10
Distance to nearest smelter [km; GM (IQR)]	10.8 (9.1–12.7)	1.2 (0.7–1.6)##	10.8 (9.1–12.7)	1.1 (0.7–1.6)##	10.7 (9.0–12.7)	1.2 (0.7–1.6)##
Smokers [no. (%)]	177 (37.0)	186 (39.0)	110 (29.8)	124 (33.8)	94 (36.4)	83 (40.9)**
Alcohol drinkers [no. (%)]	80 (17.4)	88 (18.5)	76 (20.6)	71 (19.4)	2 (0.8)	0 (0)
Serum total cholesterol [mmol/L (mean ± SD)]	6.02 ± 1.41	5.84 ± 1.37	5.68 ± 1.09	5.80 ± 1.10	5.66 ± 0.99	5.39 ± 0.98#
Serum HDL cholesterol [mmol/L (mean ± SD)]	1.42 ± 0.43	1.13 ± 0.36	1.33 ± 0.37	1.29 ± 0.36	1.46 ± 0.42	1.40 ± 0.38
Serum creatinine [μmol/L (mean ± SD)]	98 ± 24	101 ± 18	92 ± 21	89 ± 18	88 ± 17	90 ± 17
Serum γ-glutamyltransferase [U/L; GM (IQR)]	36 (22–48)	34.4 (22–48)	25 (15–35)	29 (19–41)	21 (14–28)	22 (15–29)
Serum ferritin [pmol/L; GM (IQR)]	234 (126–444)	262 (141–510)	—	—	—	—
BCd [nmol/L; GM (IQR)]	10.6 (7.1–16.9)	11.5 (6.8–19.6)*	6.3 (4.4–10.7)	8.8 (5.3–16.0)##	7.4 (5.1–11.1)	8.2 (5.3–13.7)*
UCd [nmol/day; GM (IQR)]	7.7 (5.4–11.9)	11.7 (6.8–19.5)##	6.7 (4.4–10.4)	9.1 (5.7–14.9)##	—	—
Urinary RBP [μg/day; GM (IQR)]	123 (84–173)	136 (94–196)**	68 (46–104)	73 (49–101)	—	—
Urinary creatinine [mmol/day (mean ± SD)]	12.2 ± 3.9	12.4 ± 4.3	11.8 ± 4.0	11.3 ± 3.7	—	—

Abbreviations: —, no data; GM, geometric mean. Serum ferritin was measured only at baseline. Participants did not collect a 24-hr urine sample in 2001–2003. Significance of the difference between LEA and HEA:

* 0.10 ≤ *p* < 0.05;

** *p* ≤ 0.05;

# *p* ≤ 0.01;

## *p* ≤ 0.001.

**Table 2:** Baseline characteristics (1985–1989) of participants according to follow-up status at the end of follow-up (30 September 2007).

	Alive with at least one follow-up measurement of the internal cadmium dose		Deceased		Alive without any follow-up measurement of the internal cadmium dose	
Characteristic	LEA	HEA	LEA	HEA	LEA	HEA
No.	330	314	93	113	53	53
Women [no. (%)]	182 (50.7)	177 (56.4)	51 (54.8)	59 (52.2)	27 (50.9)	29 (54.7)
Age [years (mean ± SD)]	42.1 ± 12.8	41.2 ± 12.8	67.2 ± 12.1#	66.0 ± 12.3#	41.3 ± 14.6	43.4 ± 13.8
Body mass index [kg/m^2^ (mean ± SD)]	25.6 ± 3.8	25.9 ± 4.4	26.4 ± 4.6	27.0 ± 5.2#	25.0 ± 4.4	25.4 ± 14.3
Systolic pressure [mmHg (mean ± SD)]	125 ± 15	124 ± 14	144 ± 20#	140 ± 19#	124 ± 15	129 ± 17
Diastolic pressure [mmHg (mean ± SD)]	74 ± 9	75 ± 9	78 ± 9#	78 ± 10#	73 ± 8	78 ± 11*#
Distance to nearest smelter [km; GM (IQR)]	10.7 (9.0–12.7)	1.10 (0.69–1.57)*	10.7 (9.0–12.5)	1.21 (0.68–1.66)*#	12.0 (9.5–13.2)	1.18 (0.69–1.87)*
Smokers [no. (%)]	127 (38.5)	126 (40.1)	31 (33.3)	39 (34.5)	19 (35.9)	21 (39.6)
Alcohol drinkers [no. (%)]	58 (18.5)	57 (18.4)	12 (12.9)	20 (17.9)	10 (18.9)	11 (20.8)
Serum total cholesterol [mmol/L (mean ± SD)]	5.88 ± 1.29	5.66 ± 1.30	6.52 ± 1.66#	6.38 ± 1.45#	5.95 ± 1.42#	5.78 ± 1.61#
Serum HDL cholesterol [mmol/L (mean ± SD)]	1.44 ± 0.43	1.15 ± 0.34*	1.29 ± 0.38	1.08 ± 0.37*	1.54 ± 0.45	1.13 ± 0.34*
Serum creatinine [μmol/L (mean ± SD)]	96 ± 16	98 ± 15	110 ± 39#	109 ± 23#	95 ± 17	103 ± 18
Serum γ-glutamyltransferase [U/L; GM (IQR)]	32 (22–45)	31 (19–45)	46 (22–70)#	44 (26–54)	49 (22–70)	35 (22–51)
Serum ferritin [pmol/L; GM (IQR)]	213 (34–1,021)	236 (59–1,044)	341 (57–1,522)#	368 (80–1,252)#	205 (50–989)	249 (39–1,102)
BCd [nmol/L; GM (IQR)]	10.3 (7.1–6.9)	10.2 (6.2–18.6)	13.0 (8.9–7.8)#	16.2 (9.7–13.8)*#	8.5 (5.4–15.1)	11.1 (5.3–19.6)
UCd [nmol/day; GM (IQR)]	7.4 (2.5–11.3)	10.1 (5.9–16.5)*	9.8 (9.9–12.7)#	18.9 (11.7–29.8)*#	6.9 (4.7–11.0)	10.8 (5.2–19.1)*
Urinary RBP [μg/day; GM (IQR)]	116 (83–163)	134 (98–188)	151 (85–220)#	142 (84–209)	124 (85–189)	133 (92–212)
Urinary creatinine [mmol/day (mean ± SD)]	12.7 ± 3.9	12.9 ± 4.1	10.0 ± 3.2#	10.7 ± 4.4#	12.5 ± 4.0	12.2 ± 3.9

GM, geometric mean.

* Significantly different from LEA (*p* ≤ 0.05).

# Significantly different from participants followed up and alive on 30 September 2007 (*p* ≤ 0.05).

**Table 3:** Internal dose of cadmium at follow-up predicted from characteristics at baseline (1985–1989).

Variable	Log BCd (nmol/L)	Log BCd (nmol/L)	Log UCd (nmol/24 hr)
Follow-up period	1991–1996	2001–2003	1991–1996
No. of subjects analyzed	711	461	730
Median follow-up [years (IQR)]	5.2 (4.8–5.5)	13.4 (10.7–16.0)	5.2 (4.8–5.5)
*R*^2^	0.61	0.58	0.63
Intercept (β± SE)	0.229 ± 0.090#	0.448 ± 0.093##	0.201 ± 0.075#
Partial regression coefficients (β± SE) Being female (0,1)	−0.0068 ± 0.0188	0.0051 ± 0.0198	−0.0300 ± 0.0160
Baseline (1985–1986) value (β± SE)			
Log BCd (nmol/L)	0.681 ± 0.033##	0.540 ± 0.034##	[Table-fn tfn9-ehp-116-1620]—
Log UCd (nmol/day)	[Table-fn tfn9-ehp-116-1620]—	[Table-fn tfn9-ehp-116-1620]—	0.664 ± 0.028##
Age (+10 years)	0.052 ± 0.007##	0.044 ± 0.007##	0.031 ± 0.006##
Body mass index (+1 kg/m^2^)	−0.0009 ± 0.0020	−0.0016 ± 0.0023	0.0019 ± 0.0017
Smoking (0,1)	0.086 ± 0.021##	0.084 ± 0.022##	0.073 ± 0.015##
Log serum ferritin (pmol/L)	−0.110 ± 0.024##	−0.111 ± 0.025**	−0.040 ± 0.020*
Log distance to nearest smelter (km)	−0.144 ± 0.016##	−0.069 ± 0.016##	−0.034 ± 0.014**

—, covariable not considered in the analysis. The dependent variable is the logarithmically transformed variable reflecting the internal cadmium dose at follow-up.

Significance of the partial regression coefficients:

* 0.10 ≤ *p* < 0.05;

** *p* ≤ 0.05;

# *p* ≤ 0.01;

## *p* ≤ 0.001. Being female (0, 1) and body mass index were not selected by stepwise regression (*p* ≥ 0.22), but were forced into the models.

**Table 4:** BCd and UCd at baseline (1985–1989) as predictors of mortality in environmentally exposed subjects.

	Mortality statistics by area				Hazard ratios (95% CI) associated with a doubling of the internal cadmium dose at baseline (1985–1989)^a^			
Variable	No. of deaths (%)		Standardized rate (per 1,000 person-years)		BCd	*p*-Value	UCd	*p*-Value
Study area							
Exposure level	LEA	HEA	LEA	HEA	LEA and HEA		LEA and HEA	
No. of subjects	476	480	476	480	924		944	
No. of deaths	93	113	93	113	195		201	
Cause of death								
Total	93 (19.5)	113 (23.5)	10.2	13.1	1.25 (1.04–1.50)	0.017	1.20 (1.04–1.39)	0.014
Cardiovascular	38 (8.0)	50 (10.4)	4.4	5.9	1.20 (0.90–1.60)	0.21	1.07 (0.85–1.34)	0.56
Cardiac	23 (4.8)	33 (6.9)	2.6	3.9	1.19 (0.84–1.71)	0.33	1.05 (0.79–1.40)	0.73
Cerebrovascular	12 (2.5)	9 (1.9)	1.4	1.0	0.83 (0.46–1.49)	0.52	0.70 (0.59–0.98)	0.04
Noncardiovascular	42 (8.8)	54 (11.3)	4.8	5.9	1.33 (1.01–1.75)	0.04	1.44 (1.16–1.79)	0.0009
Cancer	21 (4.4)	33 (6.9)	2.4	3.8	1.21 (0.86–1.71)	0.27	1.43 (1.08–1.89)	0.012
Lung	4 (0.8)	13 (2.7)	0.4	1.5	0.98 (0.55–1.79)	0.99	1.62 (1.02–2.55)	0.039
Gastrointestinal	7 (1.5)	9 (1.9)	0.8	1.1	1.23 (0.66–2.32)	0.51	1.25 (0.72–2.15)	0.43
Urogenital	4 (0.8)	2 (0.4)	0.5	0.2	1.04 (0.37–2.90)	0.93	1.09 (0.42–2.83)	0.87
Other cancer	6 (1.3)	9 (1.9)	0.7	1.0	1.64 (0.84–3.21)	0.15	1.69 (0.99–2.86)	0.053
Other noncardiovascular	21 (4.4)	21 (4.4)	2.3	2.2	1.57 (1.01–2.44)	0.043	1.51 (1.07–2.14)	0.02
Violent death	1 (0.2)	4 (0.8)	0.1	0.5	1.81 (0.67–4.93)	0.24	2.65 (1.12–6.27)	0.026

The cause of death could not be ascertained in 17 participants. Death rates were standardized for sex and age (20–39, 40–59, ≥ 60 years) by the direct method.

a Hazard ratios and *p*-values were computed by Cox regression and were adjusted for sex, age, body mass index, smoking status, γ-glutamyltransferase as index of alcohol intake, and SES.

**Table 5:** BCd and UCd at baseline (1985–1989) as predictors of mortality in environmentally exposed subjects and 42 smelter workers.

	Mortality statistics by area				Hazard ratios (95% CI) associated with a doubling of the internal cadmium dose at baseline (1985–1989)^a^			
Variable	No. of deaths (%)		Standardized rate (per 1,000 person-years)		BCd	*p*-Value	UCd	*p*-Value
Study area								
Exposure level	LEA	HEA	LEA	HEA	LEA and HEA		LEA and HEA	
No. of subjects	479	519	479	519	964		986	
No. of deaths	94	131	94	131	212		220	
Cause of death								
Total	94 (11.6)	131 (25.2)	11.0	13.8	1.32 (1.11–1.56)	0.001	1.22 (1.06–1.40)	0.006
Cardiovascular	38 (7.9)	60 (11.6)	4.5	6.3	1.29 (0.99–1.67)	0.057	1.11 (0.89–1.38)	0.34
Cardiac	23 (4.8)	41 (7.9)	2.7	4.3	1.31 (0.95–1.81)	0.10	1.09 (0.83–1.43)	0.54
Cerebrovascular	12 (2.5)	10 (1.9)	1.4	1.0	0.85 (0.49–1.47)	0.57	0.61 (0.37–0.99)	0.05
Noncardiovascular	43 (8.9)	61 (11.6)	5.2	6.5	1.41 (1.10–1.80)	0.007	1.43 (1.17–1.76)	0.0007
Cancer	22 (4.6)	38 (7.3)	2.5	4.0	1.27 (0.92–1.74)	0.14	1.44 (1.11–1.88)	0.007
Lung	4 (0.8)	17 (3.3)	0.4	1.8	1.11 (0.66–1.85)	0.70	1.65 (1.08–2.50)	0.019
Gastrointestinal	7 (1.5)	10 (1.9)	0.8	1.1	1.41 (0.77–2.57)	0.27	1.33 (0.79–2.26)	0.28
Urogenital	4 (0.8)	2 (0.4)	0.5	0.2	1.02 (0.37–2.84)	0.96	1.05 (0.40–2.74)	0.92
Other cancer	7 (1.5)	9 (1.7)	0.7	1.0	1.58 (0.84–2.98)	0.16	1.57 (0.93–2.64)	0.09
Other noncardiovascular	21 (4.4)	23 (4.4)	2.4	2.0	1.65 (1.11–2.45)	0.013	1.46 (1.04–2.04)	0.03
Violent death	1 (0.2)	4 (0.8)	0.1	0.5	1.75 (0.64–4.80)	0.27	2.64 (1.06–6.56)	0.026

The cause of death could not be ascertained in 17 participants. Death rates were standardized for sex and age (20–39, 40–59, ≥ 60 years) by the direct method.

a Hazard ratios and *p*-values were computed by Cox regression and were adjusted for sex, age, body mass index, smoking, γ-glutamyltransferase as index of alcohol intake, and SES.
